# Supplementary material for: Evolution and control of the phase competition morphology in a manganite film
Source: Nat Commun. 2015 Nov 25;6:8980. doi: 10.1038/ncomms9980 (PMC4674821; doi:10.1038/ncomms9980)
Supplement: Supplementary Information — Supplementary Figures 1-8 [file ncomms9980-s1.pdf]

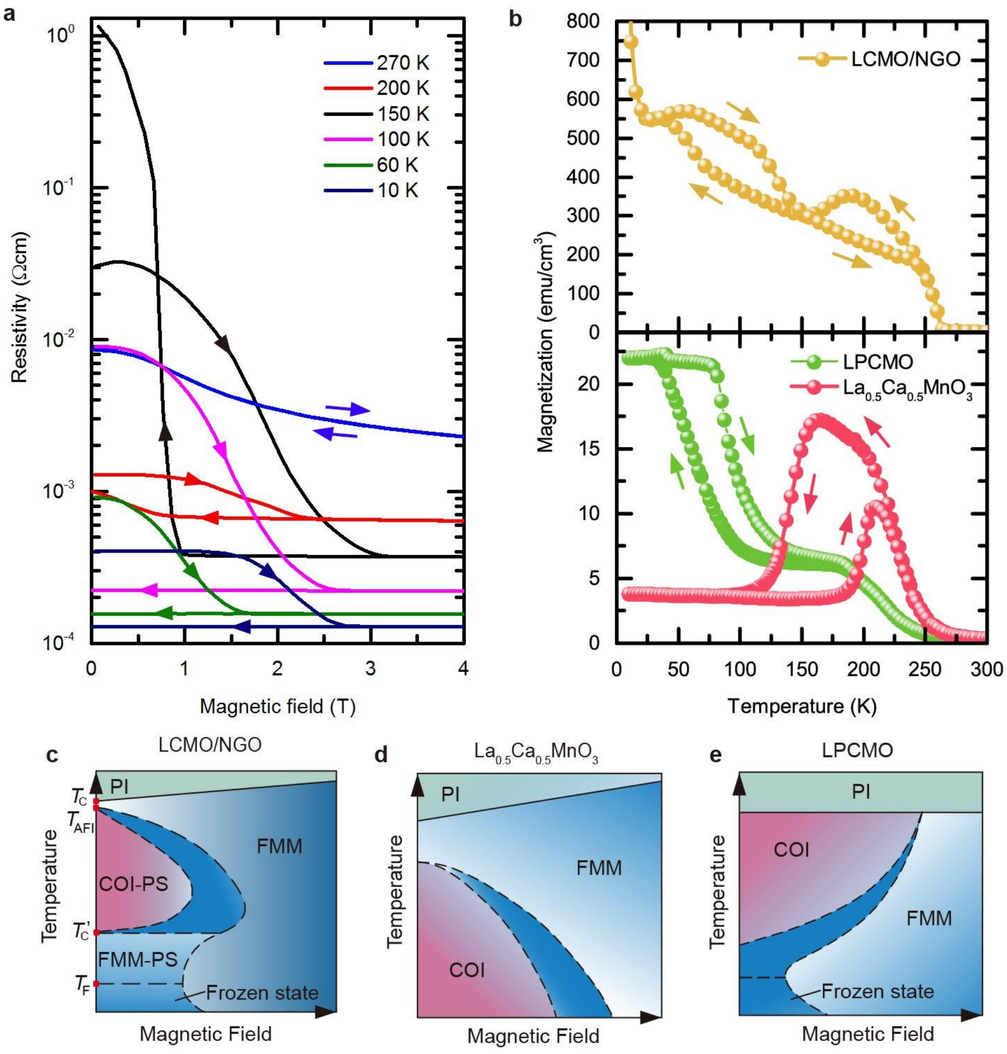

Supplementary Figure 1 | *ρ-H*, *M-T* curves and schematic *T-H* phase diagrams. (**a**) The *ρ-H* curves at various temperatures measured along the *b*-axis of the LCMO/NGO film with the magnetic field being perpendicular to the sample surface. For simplicity, only *ρ-H* curves at certain temperatures are shown**.** (**b**) The *M-T* curves of the film (top), $La_{0.5}Ca_{0.5}MnO_3$ and $La_{3/8-y}Pr_yCa_{3/8}MnO_3$ (y = 0.375) powders (bottom) at $\mu_0 H$ = 0.1 T, which are two prototype manganite PS systems. (**c**)-(**e**) Schematic phase diagrams of the three manganite PS systems. The FMM-to-COI and COI-to-FMM transitions at T_{AFI} = 250 K and T_C' = 118 K, respectively, can be seen as a combination of the FMM-to-COI transition in $La_{0.5}Ca_{0.5}MnO_3$ and COI-to-FMM transition in LPCMO.

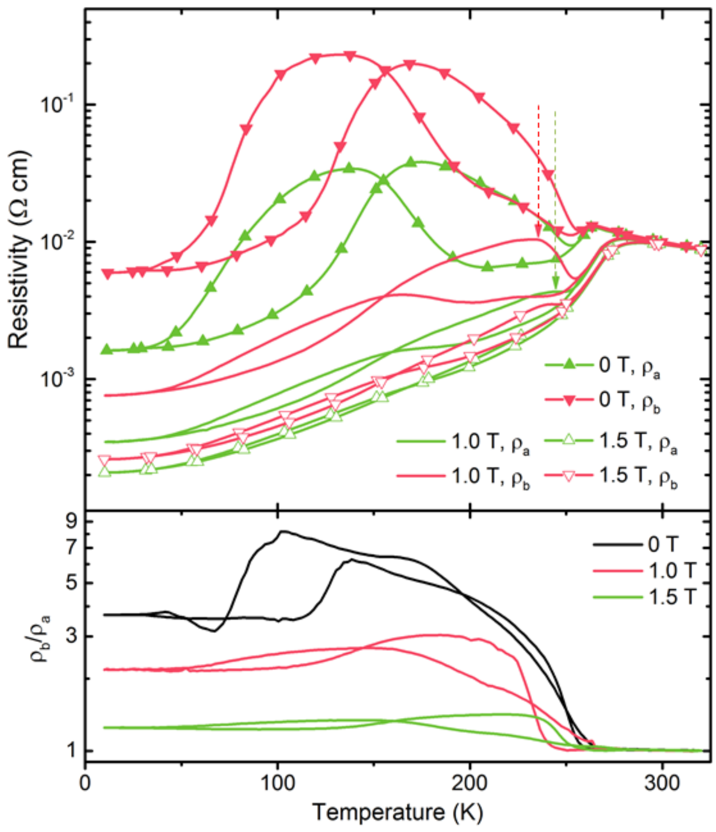

Supplementary Figure 2 | Resistivity Anisotropy. *ρ*-*T* curves along the *a*- and *b*-axis (top) and resistivity anisotropy (bottom) of a 40-nm LCMO/NGO film. The field is perpendicular to the sample surface. It is clear that below T_{AFI} = 250 K the resistivity along the *a*-axis is smaller than that along the *b*-axis. A close-up of the 1.0 T resistivity curves indicates the metal-insulator transition temperatures during warming are quite different: 245.1 K along the *a*-axis and 232.5 K along the *b*-axis, as denoted by the arrows. The FMM conducting paths along the *a*-axis during melting qualitatively account for this anisotropy.

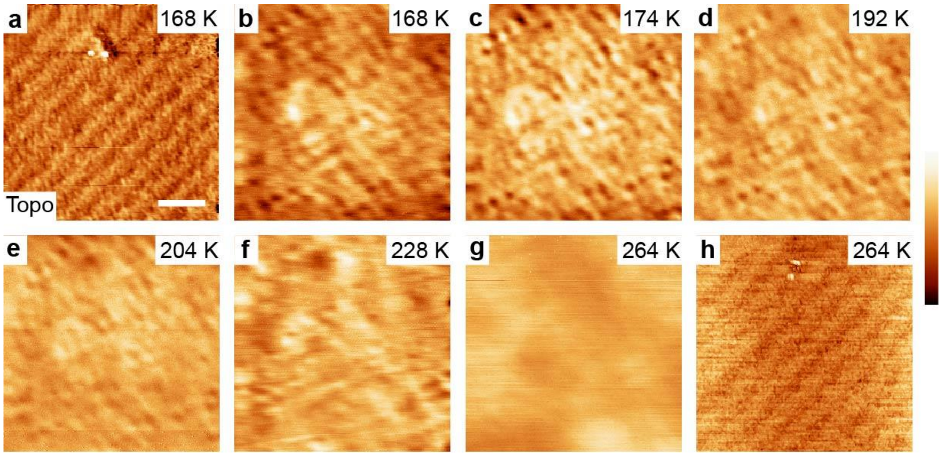

Supplementary Figure 3 | Topography and magnetic images at the same location. (**a**) The topography taken at 168 K (prior to **b**) with contact mode. (**b**)-(**h**) The MFM images during warming after the sample was cooled from room temperature to 77 K. **h** was taken with the same conditions of **g** except that the lift-height of the cantilever was reduced. Only in this situation the MFM image exhibits topographic features (the ripples). The topographic features do not change with the temperature or magnetic field. The scanned areas are 4.6 μm × 4.6 μm for all images. The color scales are 15 nm for **a** (topography)**,** 50 mHz for **b-g,** and 100 mHz for **h**.

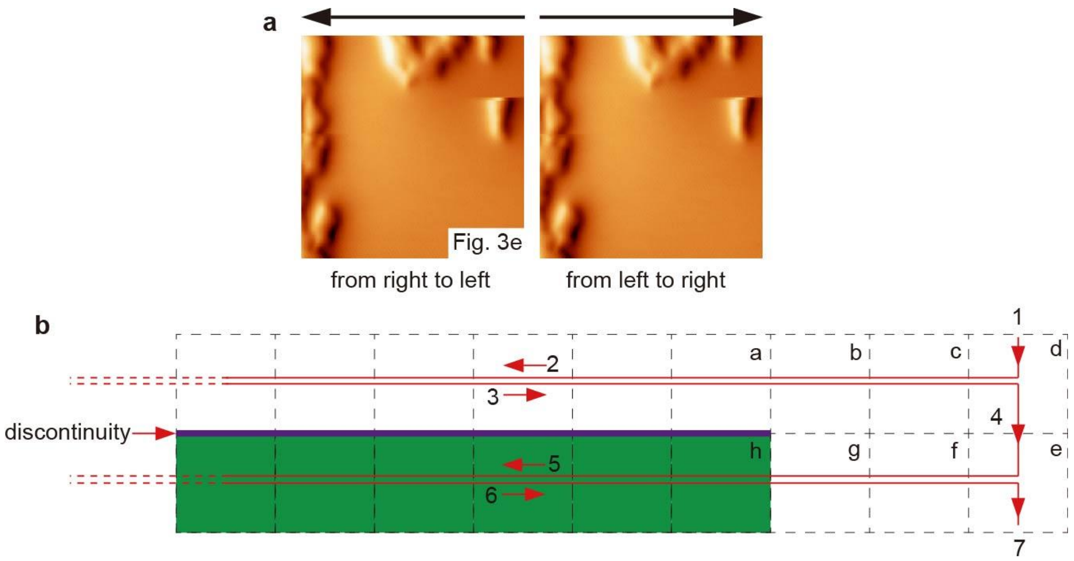

Supplementary Figure 4 | Calculation of the growth time of a COI stripe. (**a**) The images collected along opposite fast scan directions of the tip. The fast scan direction is from right to left for the left image, and from left to right for the right image. The two images are nearly the same except that there is a very small shift in the fast scan direction. Note that the bright contrast is always on the left side, independent of the fast scan direction. (**b**) Schematic of how the upper bound of the growth time is calculated. The gray dashed boxes label the individual pixels, and the red lines are the scan trajectory, i.e., the tip follows the sequence 1-2-3-4-5-6-7. The green area represents a growing COI phase domain and the deep purple line represents the sharp discontinuity. The COI domain does not appear when the tip reaches pixel a along scan line 3, but it appears when the tip reaches pixel h along scan line 5. The duration in between is the time it takes for the tip to pass seven pixels (b, c, d, e, f, g and h). It is clear that the discontinuity of a stripe domain located near the right or left edge of an image, as in the case of Fig. 3e, can give the least upper bound of the growth time. For the above image pair in **a**, this duration contains 19 pixels (the left image will give less pixels due to the aforementioned shift, ~ 3 pixels). There are 256 pixels each line and the line scan time is 0.8 s. Thus, the 19 pixels correspond to (19/256) × 0.8 s ≈ 60 ms, so the COI domain comes out within 60 ms.

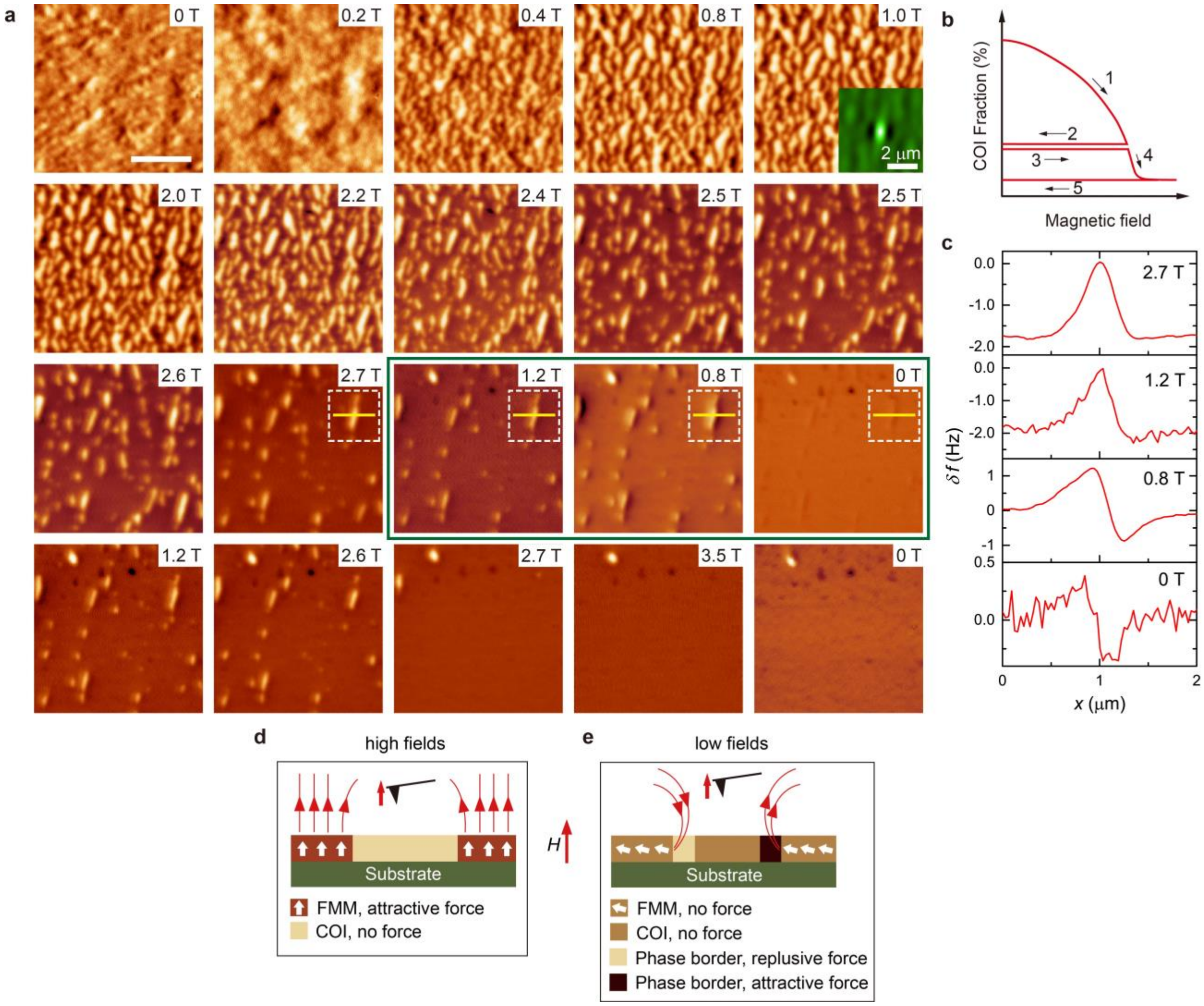

Supplementary Figure 5 | MFM images during field-sweep at 10 K and the contrast interpretation. (**a**) After zero-field cooling the sample from room temperature to 10 K, the MFM images are obtained following the field-sweep route shown in **b**. The field is applied perpendicular to the film plane. The scale bar is 2 µm and the scanned areas are 5.6 µm × 5.6 µm. The COI domains look like nematic liquid crystals, especially at fields between 0.8 T and 2.4 T. The inset in the image at 1.0 T is an auto-correlation image, showing an elongated droplet feature. The COI domains shrink from the phase domain borders when the applied field is increased. After the field reaches 2.7 T, the melting is interrupted by reducing the field to zero (path 2). Below 0.8 T, each COI domain is in bright (left side) and dark (right side) pair. This is because the magnetization of the FMM phase rotates from out-of-plane to in-plane (*b*-axis) gradually. The contrast becomes very weak when the field is further reduced to zero. It is worth mentioning

that for the images at 2.7 T to 0 T and to 2.6 T during the paths 2 and 3, the sizes of the COI domains and their distribution are almost identical, implying that the interface structural motion freezes at this state. (**c**) The cross sections along the yellow lines in the images at 2.7, 1.2, 0.8 and 0 T. One peak is seen at 2.7 T and the corresponding COI domain is purely bright. At 0.8 T, it becomes a pair of peak and valley and the corresponding COI domain is in bright-dark pair, which is consistent with in-plane magnetization (see the discussion below). (**d**)-(**e**) Interpretation of image contrasts in high and low fields, respectively. In a high field, the magnetic moments in the FMM phase are aligned with the external field. The magnetic force on the tip from the FMM domain is attractive (resonant frequency shift $\delta f < 0$). There is no force between the COI domains and tip ($\delta f = 0$), thus the COI domains in an FMM background show bright. When the field is very low, the magnetic moments in the FMM domains are mostly in-plane and the force gradient in *z*-direction (normal to the film plane) is small (the z component of the field, $B_z \approx 0$) except near the phase domain boundaries. Thus, the central area of an FMM or COI domain shows the same contrast ($\delta f = 0$, grey), while the opposite boundaries (along the *b*-axis) of an FMM or COI domain show opposite contrasts: bright ($\delta f > 0$) and dark ($\delta f < 0$).

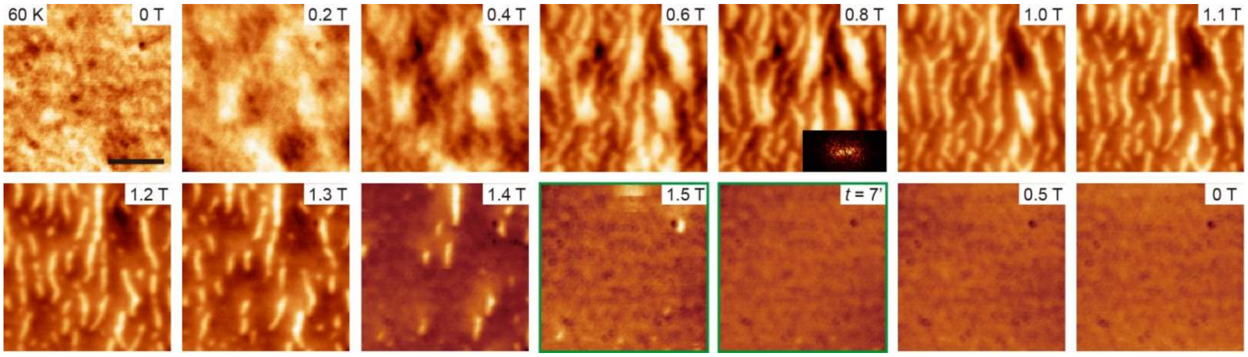

Supplementary Figure 6 | MFM images during field-sweep at 60 K. After zero-field cooling the sample from room temperature to 60 K, the MFM images are obtained. The field is applied perpendicular to the film plane. The scale bar is 2 μm and the scanned areas are 6 μm × 6 μm for all the images. The COI phase starts to melt even below 0.8 T. After the COI phase is totally melted at 1.5 T, it does not reappear when the field is reduced (similar to the case at 10 K).

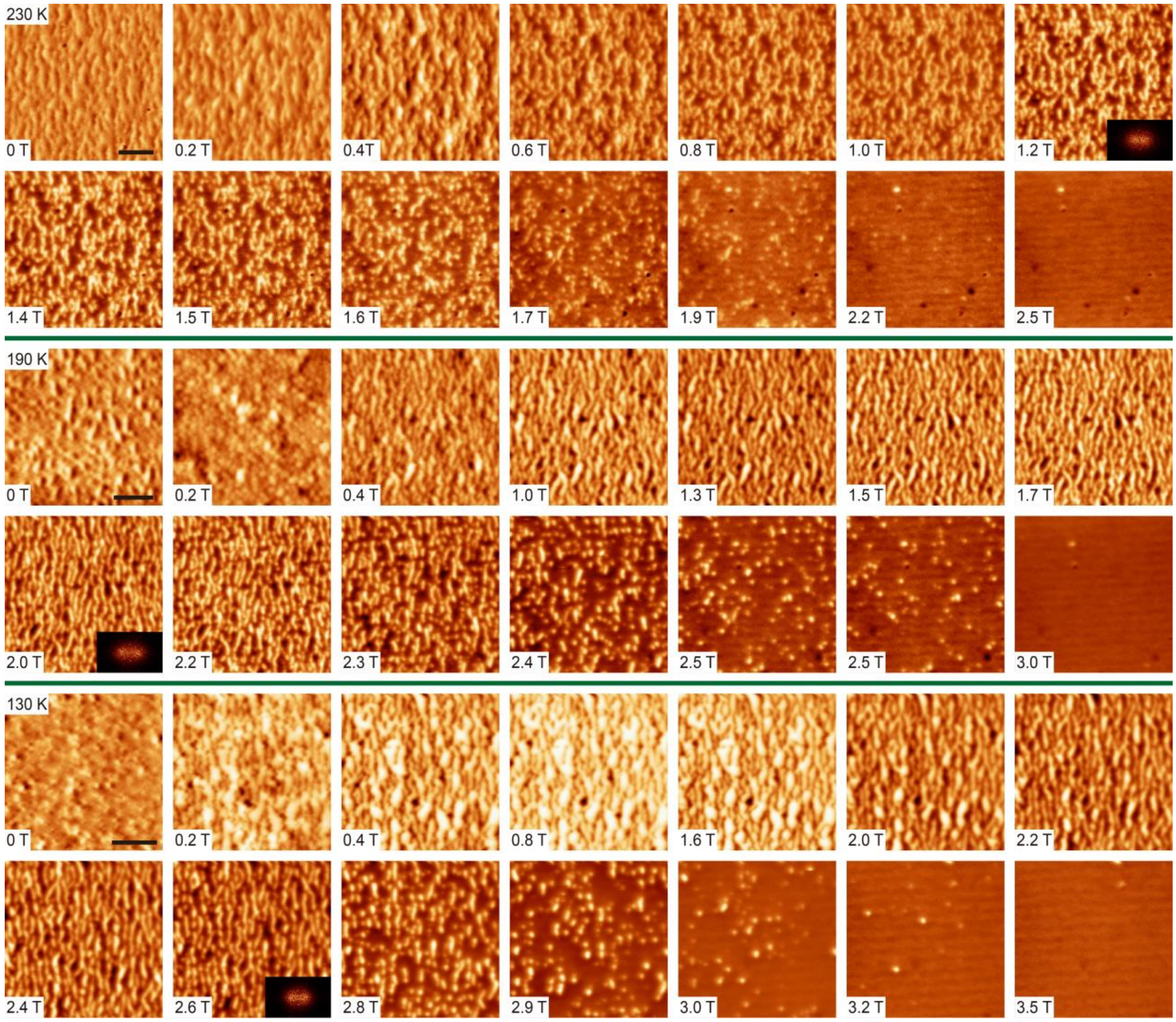

Supplementary Figure 7 | MFM image series for COI melting at 230 K, 190 K and 130 K. The sample is zero-field-cooled from room temperature to the desired temperature prior to all MFM measurements with the field being perpendicular to the film plane. The scale bars are 2 μm and the scanned areas are 9.0 μm × 9.0 μm, 8.0 μm × 8.0 μm and 7.0 μm × 7.0 μm at these three temperatures, respectively. The insets in 230 K (1.2 T), 190 K (2.0 T) and 130 K (2.6 T) are the FFT patterns, confirming the anisotropy in the corresponding images. Generally, the COI melting processes at these temperatures show similar behavior, but the fields required to fully melt all the COI phase are different, which are 2.5 T, 3.0 T and 3.5 T, respectively, implying that the COI becomes enhanced with decreasing temperature in the COI-PS regime.

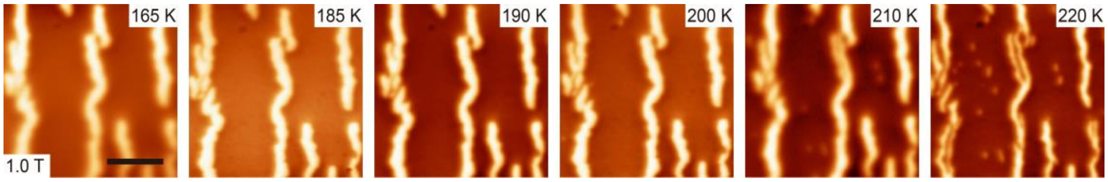

Supplementary Figure 8 | Evolution of COI domains with increasing temperature. Prior to this MFM imaging, the sample is firstly zero-field-cooled from room temperature to 165 K, and then the perpendicular magnetic field is increased to 4.0 T and decreased to 1.0 T. With the field fixed at 1.0 T, the COI stripes remain stable from 165 K to 200 K. Above 210 K, these stripes start to split from the inside and at the same time additional small droplets appear. The scale bar is 2 μm and the scanned areas are 6.4 μm × 6.4 μm.
